# A SaTScan™ macro accessory for cartography (SMAC) package implemented with SAS^® ^software

**DOI:** 10.1186/1476-072X-6-6

**Published:** 2007-03-06

**Authors:** Allyson M Abrams, Ken P Kleinman

**Affiliations:** 1 Department of Ambulatory Care and Prevention, Harvard Medical School and Harvard Pilgrim Health Care, Boston, USA; 2 Center for Education and Research on Therapeutics (CERT), HMO Research Network, Boston, USA

## Abstract

**Background:**

SaTScan is a software program written to implement the scan statistic; it can be used to find clusters in space and/or time. It must often be run multiple times per day when doing disease surveillance. Running SaTScan frequently via its graphical user interface can be cumbersome, and the output can be difficult to visualize.

**Results:**

The SaTScan Macro Accessory for Cartography (SMAC) package consists of four SAS macros and was designed as an easier way to run SaTScan multiple times and add graphical output. The package contains individual macros which allow the user to make the necessary input files for SaTScan, run SaTScan, and create graphical output all from within SAS software. The macros can also be combined to do this all in one step.

**Conclusion:**

The SMAC package can make SaTScan easier to use and can make the output more informative.

## Background

Many health departments have disease surveillance systems in place in order to detect outbreaks of illness. Other issues of public health concern may also cluster geographically. For example, vaccine or drug adverse events may cluster, due to bad manufacturer's lots or due to localized misuse/abuse. Other examples include cancers clustering due to environmental exposures.

While traditional disease surveillance involves reporting by physicians of certain lab-confirmed diseases or specific diagnoses to local, state, or national departments of health, new surveillance systems have been developed in many places which may allow detection of clusters of illness even before diagnoses have been confirmed [1-4]. This type of surveillance uses absenteeism reports, over-the-counter pharmaceutical sales, ambulatory care visits, or other data sources to determine whether there are clusters of illness [5-8].

The data for these systems are typically collected every day in an automated way and analyzed for new clusters of illness. In many settings, the surveillance data contain simple counts of cases in each zip code. These data must then be analyzed along with the historical data that have been collected on previous days. The results of analysis may be investigated by public health officials to determine whether there are any outbreaks of disease. The type of data in this kind of surveillance often includes information about the location of the cases [9].

Analysis of surveillance data that includes geographic information such as an address, census tract, or zip code is not a fully developed area. One approach to surveillance when spatial (geographic) data are available is the scan statistic [10]. This is probably the most commonly used approach in spatial disease surveillance. One reason that the scan statistic is so often used is the free availability of SaTScan [11], a software program that implements it. Although the program has a smooth graphical user interface (GUI) and accepts user input for numerous adjustable options, SaTScan can be quite cumbersome to run frequently via its GUI.

In surveillance applications, SaTScan may be run once per day for many diseases, for multiple data sources. For example, if doing daily surveillance in 2 different data sources for 10 diseases, SaTScan would need to be run 20 times per day. Usually, the user's SaTScan options remain the same for each run; it is only the input data and the destination file for the SaTScan output that change. Running SaTScan through its GUI is not the easiest or fastest way to do this repeated scanning. This problem is exacerbated for evaluation of systems that use SaTScan since evaluation of such systems may require running SaTScan hundreds or even thousands of times.

Even though the clusters discovered through SaTScan are typically in a subset of the scanning area, SaTScan lacks cartographic output. The user submits geographical data by assigning identifiers to locations; the program outputs a list of locations included in the most unusual cluster, as well as a center and radius for the cluster. While a map is not required for the output to be useful, many users probably use the SaTScan results to imagine or draw the cluster on a map.

The SaTScan Macro Accessory for Cartography (SMAC) package presented here consists of four SAS (SAS Institute, Cary, NC) macros and was designed as an easier way to run SaTScan multiple times and to add graphical output. The first macro takes as input SAS data sets in a simple prescribed format and generates text files in SaTScan format. The second macro allows the user to choose the SaTScan analysis options. The third macro reads the results from SaTScan and creates a graphical output page, based on a separate map boundary file. These 3 macros can be used individually or combined as in the fourth macro, which allows the user make the SaTScan data sets, run SaTScan, and create the output page all in one step. The SMAC package can make SaTScan easier to use and makes the output more informative by creating a map of the most unusual cluster.

### The scan statistic

The scan statistic can be used for cluster detection when spatial data are available; the scan statistic has been described previously and is summarized here [10,12,13]. Suppose that a map contains *p *points and consider all circles *C*_*i,r*_, where *i *= 1,..., *p *indicates which point is the center of the circle, and the radius, *r*, ranges from 0 to some maximum radius. When the number of cases is assumed to follow a Poisson model, then for circle *C*_*i,r*_, the likelihood ratio is

Li,r=(ni,rμi,r)ni,r(N−ni,rN−μi,r)N−ni,r
 MathType@MTEF@5@5@+=feaafiart1ev1aaatCvAUfKttLearuWrP9MDH5MBPbIqV92AaeXatLxBI9gBaebbnrfifHhDYfgasaacH8akY=wiFfYdH8Gipec8Eeeu0xXdbba9frFj0=OqFfea0dXdd9vqai=hGuQ8kuc9pgc9s8qqaq=dirpe0xb9q8qiLsFr0=vr0=vr0dc8meaabaqaciaacaGaaeqabaqabeGadaaakeaacqWGmbatdaWgaaWcbaGaemyAaKMaeiilaWIaemOCaihabeaakiabg2da9maabmaabaWaaSaaaeaacqWGUbGBdaWgaaWcbaGaemyAaKMaeiilaWIaemOCaihabeaaaOqaaGGaciab=X7aTnaaBaaaleaacqWGPbqAcqGGSaalcqWGYbGCaeqaaaaaaOGaayjkaiaawMcaamaaCaaaleqabaGaemOBa42aaSbaaWqaaiabdMgaPjabcYcaSiabdkhaYbqabaaaaOWaaeWaaeaadaWcaaqaaiabd6eaojabgkHiTiabd6gaUnaaBaaaleaacqWGPbqAcqGGSaalcqWGYbGCaeqaaaGcbaGaemOta4KaeyOeI0Iae8hVd02aaSbaaSqaaiabdMgaPjabcYcaSiabdkhaYbqabaaaaaGccaGLOaGaayzkaaWaaWbaaSqabeaacqWGobGtcqGHsislcqWGUbGBdaWgaaadbaGaemyAaKMaeiilaWIaemOCaihabeaaaaaaaa@5CA1@

where *n*_*i,r *_is the number of cases inside *C*_*i,r*_, *N *is the total number of cases, and *μ*_*i,r *_is the expected number of cases inside *C*_*i,r*_. Note here that the expected number of cases inside the circle is the total number of cases, *N*, times the proportion of the population inside the circle. In practice, we need only consider values of *r *for which *C*_*i,r *_contains a distinct set of points, since 2 circles with different radii but containing the same set of points will have the same likelihood ratio. The likelihood ratio is calculated for each circle; the *scan statistic *is the maximum likelihood ratio over all distinct circles and corresponds to the most likely cluster.

This can easily be extended to a space-time scan statistic. To do this, we turn each circle into a cylinder by giving the circle a height that represents time. The height of the cylinder varies to scan for different length clusters. The space-time scan considers all possible circles for the base of the cylinder and all possible heights, ranging from a single time period to some maximum number of time periods. The likelihood ratio is calculated for each cylinder and the scan statistic is the maximum likelihood ratio over all possible cylinders, again corresponding to the most likely cluster.

A p-value is calculated for the scan statistic using Monte Carlo hypothesis testing [10]. This process has several steps. In the first step, the proportion of the population living at each point is calculated. Second, a Monte Carlo replicate is created by assigning each case to a point, with the probability for assignment to a point equal to the population proportion at that point. Next, the scan statistic for the Monte Carlo replicate is calculated. These steps are repeated *M *times; the p-value is k+1M+1
 MathType@MTEF@5@5@+=feaafiart1ev1aaatCvAUfKttLearuWrP9MDH5MBPbIqV92AaeXatLxBI9gBaebbnrfifHhDYfgasaacH8akY=wiFfYdH8Gipec8Eeeu0xXdbba9frFj0=OqFfea0dXdd9vqai=hGuQ8kuc9pgc9s8qqaq=dirpe0xb9q8qiLsFr0=vr0=vr0dc8meaabaqaciaacaGaaeqabaqabeGadaaakeaadaWcaaqaaiabdUgaRjabgUcaRiabigdaXaqaaiabd2eanjabgUcaRiabigdaXaaaaaa@32E2@, where *k *is the number of Monte Carlo scan statistics greater than or equal to the scan statistic in the real data. P-values for secondary clusters can be found in a similar fashion by considering the second, third, etc. largest likelihood ratios based on the real data.

In many surveillance systems, cases are aggregated so that every case within a region is considered to be located at the centroid of that region. In this case, if the centroid of the region is in the circle, then all cases from the region are included in that circle. If the centroid is not in the circle, then none of the cases from that region will be included. Cases may be aggregated by zip code, for example, because the data come with a zip code for each case, but not an exact address, or to protect privacy of the individuals under surveillance [14].

### SaTScan

SaTScan is a freely available software program created to implement the scan statistic [11]. There are two data sets that are required in order to run SaTScan and several optional files. One required data set is a coordinates file containing location identifiers (IDs) and x and y coordinates describing the location. The other required file is a case file which contains location IDs, number of cases, and date.

There are also several optional input files. The most common of these is a population file which is required to calculate the likelihood ratio with a Poisson model. This file contains the location ID, date, and population in that location ID on that date. A Bernoulli model would necessitate a control file instead of a population file; this file would have exactly the same format as the population file, but would contain the number of controls for each location ID instead of the population. Other models can also be used in SaTScan, but the current version of the SMAC package only allows the user to choose the Poisson model.

The typical user must first put the data in the correct format for SaTScan, open the SaTScan GUI, enter the file names and locations, choose the appropriate options by clicking through a series of tabs, and run SaTScan to find the most likely clusters and p-values based on the scan statistics. Assuming there are no errors, the SaTScan output consists of one or more text files with statistical and geographical information about the clusters. These files can be viewed in SaTScan or may be opened in any text editor. The files contain a list of location IDs included in the cluster along with some statistics about the cluster, including the number of observed cases, the number of expected cases, the log likelihood ratio, and the p-value for the cluster. See additional files 1, 2, 3 for sample SaTScan output. There is no graphical output included. This means that the user must figure out where each location ID is on a map. If, for example, the output contains a list of zip codes, then the user must figure out where those zip codes are.

Some common errors in the input files include a case file that contains cases on dates outside of the date range specified for analysis, or cases in a location ID that is not included in the coordinates file. These types of errors will cause SaTScan to stop running and require the input files to be fixed.

## Results

The SMAC package consists of four SAS macros designed to address some of the difficulties in running SaTScan through its GUI. One macro creates the input files in SaTScan format, a second macro allows the user to choose the SaTScan options, and the third macro reads in the SaTScan output and combines it with boundary files in order to create a one-page summary of the most unusual cluster including a map of the cluster and some key statistical information about the cluster as well as a list of the locations within the cluster. The boundary files must be obtained from another source. The US Census Bureau has boundary files available for various geographical areas [15,16]. The fourth macro in the SMAC package calls each of the first 3 macros in order to create the input files, run SaTScan, and create the graphical output all in one step.

Below is a detailed description of each macro. The code for these macros can be found in additional files 4, 5, 6, 7. Note that in their current form, the macros require 5-digit zip codes as location IDs. Also note that minor modifications are needed to use the SMAC package with some scan options.

### The "inputdata" macro

The *inputdata *macro makes data sets in the format required by SaTScan. As input, this macro requires three SAS data sets as well as the number of days in the study period, the last date of the study period, and the names and locations where the output files will be stored. The macro generates text files in SaTScan format as output. The SAS data sets must all be stored in the same folder, which must also be specified in the macro call. If any of the SAS data sets are missing or any of the required variables are missing the macro will write an error in the log and SAS will stop.

One required SAS data set contains the location information. Variables must include location ID and x and y coordinates for the location. The coordinates can be latitude/longitude pairs or Cartesian coordinates. From this data set, the *inputdata *macro will create the coordinates file to be used in SaTScan.

A second SAS data set contains variables for the location ID, the date of the reported cases as a SAS date, and the number of cases. The *inputdata *macro will make the case file in the correct format for SaTScan using only the cases that occur within the given time period and in a location ID that is in the coordinates file. Note that this last feature prevents one of the common SaTScan errors, discussed above.

Finally, there must be a SAS data set containing population data with either 2 or 3 variables. Only location IDs that are in the coordinates file are included in the generated population file. If the population changes over time, then the data set must include the location ID, date, and population on that date. If the population is constant during the analysis period, only the location ID and population are required. If there is no date included in the data set, a warning will appear in the log, for example:

WARNING: The required variable "date" was not found in the data set "a.population_sas". It will be assumed that the population is the same every day in each location ID.

If any of the three SAS data sets is missing, an error message will appear in the SAS log and the macro will stop running. For example, if the data set containing case information is supposed to be stored in the SAS data set called "a.cases_sas" but does not exist, the following message will appear in the log, for example:

**ERROR: The SAS data set "a.cases_sas", which should contain the number of cases for each location ID/date pair, does not exist. The INPUTDATA macro will stop running**.

If the requisite variables do not exist within the data sets, an error message will appear in the SAS log so that the user will be able to tell which variable is missing, and from which data set.

If all of the data sets and variables exist, the SaTScan input files will be created as described above. If a file does not exist, an error message will appear in the log.

### The "parameters" macro

The *parameters *macro allows the user to specify all of the SaTScan options. The input for this macro includes the names and locations of all of the input and results files for SaTScan. All of the options available through the SaTScan GUI can also be specified here, including the study period, the type of analysis to be done (purely temporal, purely spatial, or space-time) and whether the analysis should be retrospective (scanning over the entire study period) or prospective (scanning for clusters which include the study end date only). The output from this macro is a text file containing all of the necessary SaTScan input parameters in the correct format to be used by SaTScan. This file is stored in two places – in the directory where SaTScan is installed on the user's hard drive and in the folder on the hard drive where the SaTScan results are to be stored. A warning is written in the SAS log if the files do not exist.

Note that we expect that this macro will be used in conjunction with the SMAC macro, described below. If running the *parameters *macro independently, the user must enter every SaTScan option. In the SMAC macro, there are defaults set for most SaTScan options, so the user only needs to specify options that are different from the defaults.

### The "map" macro

The *map *macro uses the results from SaTScan and outputs a graphical page with the most likely cluster of zip codes highlighted in color along with some key information about the cluster. The inputs to this macro are the location of the SAS data sets containing the mapping data, the names of the required SAS data sets (one containing the centroid for each location and the other containing the boundary information for each location), the name and location of the results files from the SaTScan analysis, the name and location of the file that will be created using the *map *macro, and the start and end dates of the analysis period. In the current version of the *map *macro, we use zip code centroids and boundaries from the 2000 census [16]. Additionally, a data set containing location IDs and location names can be included, which allows the name of the location of the cluster center to be printed on the output page.

Running SaTScan should result in the creation of at least three output files. These files should all be stored in the same folder on the hard drive, and the names of these files will be the same except for the extension. If any of these three files are missing, the macro will print an error message similar to the error message from the *inputdata *macro and SAS will stop. Otherwise, the macro will create a map with the zip codes in the most likely cluster indicated.

The most likely cluster is indicated on the map by coloring in the included zip codes. The zip code at the center of the cluster is colored red, while all other zip codes in the cluster are shaded gray. There is red text describing the cluster; it contains the study dates, the cluster dates and length of the cluster, the name of the city where the cluster is centered, the zip code of the cluster center, the radius of the cluster, the number of observed and expected cases, and the p-value of the cluster. There is also black text which lists all of the zip codes in the cluster.

After running the macro, the map can be viewed by opening the graph window in SAS. The output is also stored as a computer graphics metafile (CGM) in a location indicated in the macro call. CGM formatted files can be inserted as a picture into Microsoft Word or PowerPoint documents. The macro checks to make sure that the CGM file exists, and writes an error message in the log otherwise.

### The "SMAC" macro

The *SMAC *macro incorporates the other 3 macros. First, this macro calls the *inputdata *macro. Then, a check is run to make sure that the date specified as the starting date for analysis is before the last date. If not, an error message will appear in the log.

If no errors are found, then the *parameters *macro is run. The *SMAC *macro automatically uses the same file names in the *parameters *macro that were created in the *inputdata *macro so that these only need to be entered once. Also, most of the SaTScan options have defaults set in the *SMAC *macro so that the user need only change the settings that are different from the defaults, instead of having to enter every single option. Some of the defaults include coordinate units (defaulting to latitude/longitude), type of analysis (prospective space-time), maximum cluster size (50% of population at risk), maximum cluster length (3 days), and number of Monte Carlo repetitions (999).

After the *parameters *macro runs successfully, the *SMAC *macro calls SaTScan, which runs in batch mode. Finally, the *map *macro runs to create the graphical output described above.

### Example

We used the space-time scan statistic to analyze reports of cryptosporidium from the Massachusetts Department of Public Health (MDPH) as an example here. The original data set included all cases reported to the MDPH during 2002; the analysis was done on approximately 6 months of data, from March 12, 2002 through September 9, 2002. The clusters in this analysis were allowed to be geographically large enough to include up to 50 percent of the population of Massachusetts and be up to 14 days long. Sample output from the macros included in the SMAC package are described below.

The case data originated in a Microsoft Excel spreadsheet. After importing into SAS and putting into the correct SAS format, this can be used as input in the *inputdata *macro. Note that there are not cases in every zip code/day, so there may be zip codes and/or dates that do not appear in the case file that do exist in the other files. This is not problematic; in SaTScan such absences are treated as data showing no cases for those zip code/days. Shown in table 1 are the first 5 lines from each of the files that the *inputdata *macro creates.

**Table 1:** Sample data sets produced by *inputdata *macro, to be read into SaTScan

Case file			Population file			Coordinates file		
Zip code	# cases	Date	Zip code	Date	Population	Zip code	Latitude	Longitude

01473	1	2002/06/26	01420	2002/03/12	39100	01420	42.59501313	-71.81566156
01501	1	2002/08/23	01420	2002/03/13	39100	01430	42.660260506	-71.92062028
01534	1	2002/07/18	01420	2002/03/14	39100	01431	42.677352516	-71.81748489
01550	1	2002/08/20	01420	2002/03/15	39100	01432	42.554193068	-71.57240053
01566	1	2002/07/25	01420	2002/03/16	39100	01436	42.608953883	-72.0898413

The *parameters *macro produces a text file containing all of the options for SaTScan, in the correct format to be read into SaTScan. An example file is shown in additional file 8.

**Figure 1:**
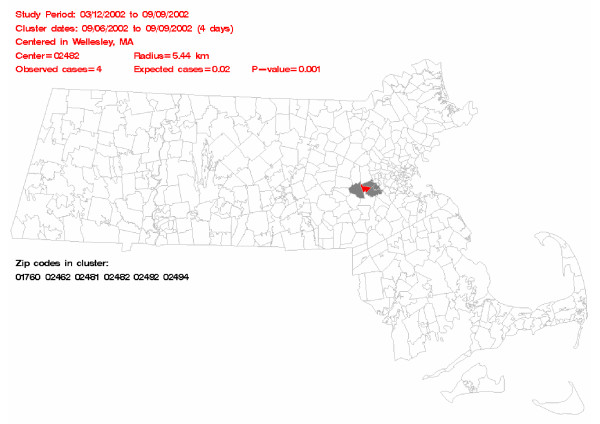
**Sample output page generated using the *map *macro**. Some key information about the most likely cluster appears in red text above the map, while the zip codes in the most likely cluster are listed below the map in black text. The zip code colored in red is the center of the most likely cluster, and the gray zip codes are the other zip codes in the most likely cluster.

Figure 1 shows an example of the output page generated by the *map *macro. Some key information about the most likely cluster appears in red text above the map, while the zip codes in the most likely cluster are listed below the map in black text. On the map, the zip code colored in red is the center of the most likely cluster, and the gray zip codes are the other zip codes in the most likely cluster.

The *SMAC *macro generates all of the above results, but in only one step, instead of having to run each macro individually. It also adds more error checks and runs SaTScan. This is not only more convenient, but also saves a great deal of time, especially when compared to running SaTScan many times manually or via the individual macros.

## Discussion

We have developed a SAS macro package to help run SaTScan in a more efficient, less tedious manner. The macros may be used separately or in combination in order to make SaTScan input files, choose SaTScan options, and view output from SaTScan. The user needs to be familiar with SaTScan and all of the options available; for those who are familiar with SaTScan and who have a little SAS knowledge, the SMAC package is an easy-to-use SAS interface which allows the user to run SaTScan from within SAS and create maps of the SaTScan cluster.

The SAS code for the SMAC package was written to work in SAS for Windows, version 8.2 and may need slight modifications with other versions. The current version of the SMAC package also only works with geographical regions that are zip codes, although this would be easy to change. In addition, we have included a data set that associates city names with zip codes. This would have to be modified for use with other types of regions. SaTScan is also in continual development. The macros presented here work with SaTScan version 5.1. As SaTScan changes, modifications to the macros will be required to enable smooth operation.

One of the restrictions of the *inputdata *macro is that it assumes that analysis will be done using a population file. In order to do other types of analyses, the macro would need to be changed. For example, to use a Bernoulli model the macro would need to make a control file instead of a population file. To use the space-time permutation scan statistic, neither population nor control data are required, so the macro would need to be changed to make only two data sets instead of three.

The *map *macro in additional file 6 is configured to work with Massachusetts zip code data. Users can easily customize the macro to use data from other regions. Another limitation of this macro is that it only creates a map of the primary cluster, the cluster found by SaTScan to be the most unusual cluster. Although other clusters may exist, the user must view the text files created from SaTScan in order to find out any information about those clusters. The macro could be modified to generate a separate map for every cluster with a p-value below a certain threshold. Finally, in order to keep this macro as simple as possible, the only text that appears on the map is that found in the SaTScan output files. Since the disease being analyzed does not appear in these files anywhere, there is currently no text on the map that indicates the disease. An option to include this and other additional text may be added in the future.

One advantage of the SMAC package is that any parameter that is allowable in SaTScan can be accessed and modified via the *parameters *macro. This includes parameters that are allowed only in batch mode and can be easily extended when new options arise in future releases. One potential example of this is the flexibly shaped scan statistic proposed by Tango and Takahashi [17].

Another advantage of the *SMAC *macro is that it is quite simple to write another SAS macro that calls the *SMAC *macro repeatedly over a period of time by using a simple do loop with the date as the index. The *SMAC *macro can then be called using the date in the macro call. In this way, all of the files can easily be managed by including the date in the file name, none of the files need to be created manually, and SaTScan will run time after time without user intervention. See additional file 9 for a sample macro which calls the *SMAC *macro multiple times.

## Conclusion

Assuming the data reside in SAS or an easily imported format such as an EXCEL spreadsheet, the macros presented here can save steps and time for applications in which SaTScan is run frequently. The SMAC package allows the user to easily create the needed data sets and run SaTScan within SAS, and provides graphical output of the likely cluster.

## Competing interests

The author(s) declare that they have no competing interests.

## Authors' contributions

AA wrote the macros and drafted the manuscript. KK conceived of the idea for the SMAC package, provided guidance, and helped draft the manuscript.

## Supplementary Material

Additional File 1 **Sample text output from SaTScan**. This is a sample text file generated as output from SaTScan. It contains detailed information about each cluster, as well as a summary of the parameters that were used to obtain these results. Click here for file

Additional File 2 **Sample cluster output from SaTScan**. Most of the information about each cluster is available in column format from SaTScan, as seen here. Note that in the text file that SaTScan produces, there are no column headings. Click here for file

Additional File 3 **Sample GIS output from SaTScan**. SaTScan also creates a text file with mapping information for use with GIS software. Here is an example. Again, note that there are no column headings in the SaTScan output. Click here for file

Additional File 4 **SAS code for *inputdata *macro**. This section contains the SAS code for the *inputdata *macro described in this paper. Click here for file

Additional File 5 **SAS code for *parameters *macro**. This section contains the SAS code for the *parameters *macro described in this paper. Click here for file

Additional File 6 **SAS code for *map *macro**. This section contains the SAS code for the *map *macro described in this paper. Click here for file

Additional File 7 **SAS code for *SMAC *macro**. This section contains the SAS code for the *SMAC *macro described in this paper. Click here for file

Additional File 8 **Parameter file generated by *parameters *macro**. This is an example of a typical parameter file generated from the *parameters *macro. Click here for file

Additional File 9 **SAS macro to run SMAC package**. This is a sample SAS macro which can be used to run the SMAC package many times. Click here for file
