# Supplementary material for: A SaTScan™ macro accessory for cartography (SMAC) package implemented with SAS® software
Source: Int J Health Geogr. 2007 Mar 6;6:6. doi: 10.1186/1476-072X-6-6 (PMC1821006; doi:10.1186/1476-072X-6-6)
Supplement: Additional File 1 — Sample text output from SaTScan. This is a sample text file generated as output from SaTScan. It contains detailed information about each cluster, as well as a summary of the parameters that were used to obtain these results. [file 1476-072X-6-6-S1.pdf]

```
                    _____________________________

                           SaTScan v5.1.1
                    _____________________________


Program run on: Tue Aug 23 08:17:49 2005

Prospective Space-Time analysis
scanning for clusters with high rates
using the Poisson model.
________________________________________________________________

SUMMARY OF DATA

Study period..........: 2002/3/12 - 2002/9/9
Number of locations...: 382
Total population......: 5508750
Total cases...........: 48
Annual cases / 100000.: 1.7
________________________________________________________________

MOST LIKELY CLUSTER

1.Location IDs included.: 02482, 02481, 02462, 02492, 02494,
                          01760
  Coordinates / radius..: (42.296345 N, 71.293221 W) /  5.44 km
  Time frame............: 2002/9/6 - 2002/9/9
  Population............: 88920
  Number of cases.......: 4
  Expected cases........: 0.02
  Annual cases / 100000.: 410.8
  Observed / expected...: 234.900
  Log likelihood ratio..: 18.023758
  Monte Carlo rank......: 1/1000
  P-value...............: 0.001
  Null Occurrence.......: Once in 2 years and 272 days

SECONDARY CLUSTERS

2.Location IDs included.: 01983, 01984, 01982, 01923, 01921,
                          01949, 01969, 01833, 01915, 01960,
                          01938, 01922, 01845, 01834, 01864,
                          01929, 01940
  Coordinates / radius..: (42.640877 N, 70.935860 W) / 14.30 km
  Time frame............: 2002/9/2 - 2002/9/9
  Population............: 238503
  Number of cases.......: 2
  Expected cases........: 0.09
  Annual cases / 100000.: 38.3
  Observed / expected...: 21.894
  Log likelihood ratio..: 4.302328
  Monte Carlo rank......: 421/1000
  P-value...............: 0.421
  Null Occurrence.......: Once in 2 days

3.Location IDs included.: 02364, 02367, 02332, 02338, 02330,
```

```
                          02359, 02341, 02050, 02346, 02360,
                          02366, 02333, 02339, 02324, 02382,
                          02061, 02358, 02370, 02576, 02379,
                          02571, 02302, 02351, 02767, 02538,
                          02066, 02718, 02347, 02770, 02190,
                          02558, 02301, 02343, 02532, 02025,
                          02043, 02738, 02189, 02322, 02375,
                          02188, 02780, 02779, 02743, 02717,
                          02368, 02356, 02184, 02191, 02702,
                          02072, 02559, 02739, 02045, 02542,
                          02563, 02169, 02534, 02745, 02766,
                          02715, 02764, 02021, 02537, 02644,
                          02170, 02048, 02746, 02556, 02067,
                          02186, 02720, 02171, 02726, 02747,
                          02719, 02740, 02035, 02668, 02122,
                          02126, 02124, 02032, 02744, 02062,
                          02648, 02136, 02536, 02125, 02769,
                          02657, 02723, 02121
  Coordinates / radius..: (41.983871 N, 70.741760 W) / 45.73 km
  Time frame............: 2002/9/4 - 2002/9/9
  Population............: 1471875
  Number of cases.......: 3
  Expected cases........: 0.42
  Annual cases / 100000.: 12.4
  Observed / expected...: 7.095
  Log likelihood ratio..: 3.372279
  Monte Carlo rank......: 554/1000
  P-value...............: 0.554
  Null Occurrence.......: Once in 2 days

The log likelihood ratio value required for an observed
cluster to be significant at level
... 0.01: 10.913655
... 0.05: 8.495663
```

```
PARAMETER SETTINGS

Input Files
-----------
  Case File                    : c:\SMAC\SaTScan files\cases.txt
  Population File              : c:\SMAC\SaTScan files\population.txt
  Coordinates File             : c:\SMAC\satscan files\zipcodes.txt

  Time Precision     : Day
  Coordinates        : Latitude/Longitude

Analysis
--------
  Type of Analysis    : Prospective Space-Time
  Probability Model   : Poisson
  Scan for Areas with : High Rates

  Start Date : 2002/3/12
  End Date   : 2002/9/9
```

```
  Number of Replications : 999


Scanning Window
---------------
  Maximum Spatial Cluster Size           : 50.00 %
  Also Include Purely Temporal Clusters : No
  Maximum Temporal Cluster Size          : 14.00 Days
  Also Include Purely Spatial Clusters  : No

Time Parameters
---------------
  Time Aggregation Units  : Day
  Time Aggregation Length : 1

  Temporal Adjustment : None
  Spatial Adjustment  : None
  Adjusted for Earlier Analyses : No

Output
------
  Results File          : c:\SMAC\SaTScan files\crypto output.txt
  Cluster File          : c:\SMAC\SaTScan files\crypto output.col.txt
  Location File         : c:\SMAC\SaTScan files\crypto output.gis.txt
  Simulated LLRs File   : c:\SMAC\SaTScan files\crypto output.llr.txt

  Criteria for Reporting Secondary Clusters : No Geographical Overlap
```

```
Program completed  : Tue Aug 23 08:18:16 2005
Total Running Time : 27 seconds
```
