# Supplementary material for: A SaTScan™ macro accessory for cartography (SMAC) package implemented with SAS® software
Source: Int J Health Geogr. 2007 Mar 6;6:6. doi: 10.1186/1476-072X-6-6 (PMC1821006; doi:10.1186/1476-072X-6-6)
Supplement: Additional File 2 — Sample cluster output from SaTScan. Most of the information about each cluster is available in column format from SaTScan, as seen here. Note that in the text file that SaTScan produces, there are no column headings. [file 1476-072X-6-6-S2.pdf]

Most Central Location	Cluster Number	x-coord. or latitude	y-coord. or longitude	Circle Radius	# Location IDs	Observed Cases	Expected Cases	Observed/ Expected in cluster	LLR or Test Statistic	p-value of cluster	Cluster Start Date	Cluster End Date
02482	1	42.2963	-71.2932	5.44	6	4	0.02	234.90	18.023758	0.00100	2002/9/6	2002/9/9
01983	2	42.6409	-70.9359	14.30	17	2	0.09	21.89	4.302328	0.42100	2002/9/2	2002/9/9
02364	3	41.9839	-70.7418	45.73	93	3	0.42	7.10	3.372279	0.55400	2002/9/4	2002/9/9
