# Supplementary material for: A SaTScan™ macro accessory for cartography (SMAC) package implemented with SAS® software
Source: Int J Health Geogr. 2007 Mar 6;6:6. doi: 10.1186/1476-072X-6-6 (PMC1821006; doi:10.1186/1476-072X-6-6)
Supplement: Additional File 3 — Sample GIS output from SaTScan. SaTScan also creates a text file with mapping information for use with GIS software. Here is an example. Again, note that there are no column headings in the SaTScan output. [file 1476-072X-6-6-S3.pdf]

Location ID	Cluster Number	Observed Cases in Cluster	Expected Cases in Cluster	Observed/ Expected in Cluster	p-value in in Cluster	Observed Cases in Location	Expected Cases in Location	Observed/ Expected in Location
02482	1	4	0.02	234.900	0.00100	0	0.00	0.000
02481	1	4	0.02	234.900	0.00100	2	0.00	737.131
02462	1	4	0.02	234.900	0.00100	0	0.00	0.000
02492	1	4	0.02	234.900	0.00100	0	0.00	0.000
02494	1	4	0.02	234.900	0.00100	0	0.00	0.000
01760	1	4	0.02	234.900	0.00100	2	0.01	327.717
01983	2	2	0.09	21.894	0.42100	0	0.00	0.000
01984	2	2	0.09	21.894	0.42100	0	0.00	0.000
01982	2	2	0.09	21.894	0.42100	0	0.00	0.000
01923	2	2	0.09	21.894	0.42100	0	0.01	0.000
01921	2	2	0.09	21.894	0.42100	0	0.00	0.000
01949	2	2	0.09	21.894	0.42100	0	0.00	0.000
01969	2	2	0.09	21.894	0.42100	0	0.00	0.000
01833	2	2	0.09	21.894	0.42100	0	0.00	0.000
01915	2	2	0.09	21.894	0.42100	0	0.02	0.000
01960	2	2	0.09	21.894	0.42100	1	0.02	54.248
01938	2	2	0.09	21.894	0.42100	0	0.00	0.000
01922	2	2	0.09	21.894	0.42100	0	0.00	0.000
01845	2	2	0.09	21.894	0.42100	0	0.01	0.000
01834	2	2	0.09	21.894	0.42100	0	0.00	0.000
01864	2	2	0.09	21.894	0.42100	0	0.01	0.000
01929	2	2	0.09	21.894	0.42100	0	0.00	0.000
01940	2	2	0.09	21.894	0.42100	1	0.00	226.210
02364	3	3	0.42	7.095	0.55400	0	0.00	0.000
02367	3	3	0.42	7.095	0.55400	0	0.00	0.000
02332	3	3	0.42	7.095	0.55400	0	0.00	0.000
02338	3	3	0.42	7.095	0.55400	0	0.00	0.000
02330	3	3	0.42	7.095	0.55400	0	0.00	0.000
02359	3	3	0.42	7.095	0.55400	0	0.00	0.000
02341	3	3	0.42	7.095	0.55400	0	0.00	0.000
02050	3	3	0.42	7.095	0.55400	0	0.01	0.000
02346	3	3	0.42	7.095	0.55400	0	0.01	0.000
02360	3	3	0.42	7.095	0.55400	0	0.01	0.000
02366	3	3	0.42	7.095	0.55400	0	0.00	0.000
02333	3	3	0.42	7.095	0.55400	0	0.00	0.000
02339	3	3	0.42	7.095	0.55400	0	0.00	0.000
02324	3	3	0.42	7.095	0.55400	0	0.01	0.000
02382	3	3	0.42	7.095	0.55400	0	0.00	0.000
02061	3	3	0.42	7.095	0.55400	0	0.00	0.000

02358	3	3	0.42	7.095	0.55400	0	0.00	0.000
02370	3	3	0.42	7.095	0.55400	0	0.01	0.000
02576	3	3	0.42	7.095	0.55400	0	0.00	0.000
02379	3	3	0.42	7.095	0.55400	0	0.00	0.000
02571	3	3	0.42	7.095	0.55400	0	0.00	0.000
02302	3	3	0.42	7.095	0.55400	0	0.01	0.000
02351	3	3	0.42	7.095	0.55400	0	0.00	0.000
02767	3	3	0.42	7.095	0.55400	0	0.00	0.000
02538	3	3	0.42	7.095	0.55400	0	0.00	0.000
02066	3	3	0.42	7.095	0.55400	0	0.00	0.000
02718	3	3	0.42	7.095	0.55400	0	0.00	0.000
02347	3	3	0.42	7.095	0.55400	0	0.00	0.000
02770	3	3	0.42	7.095	0.55400	0	0.00	0.000
02190	3	3	0.42	7.095	0.55400	0	0.00	0.000
02558	3	3	0.42	7.095	0.55400	0	0.00	0.000
02301	3	3	0.42	7.095	0.55400	0	0.02	0.000
02343	3	3	0.42	7.095	0.55400	0	0.00	0.000
02532	3	3	0.42	7.095	0.55400	0	0.00	0.000
02025	3	3	0.42	7.095	0.55400	0	0.00	0.000
02043	3	3	0.42	7.095	0.55400	0	0.01	0.000
02738	3	3	0.42	7.095	0.55400	0	0.00	0.000
02189	3	3	0.42	7.095	0.55400	0	0.00	0.000
02322	3	3	0.42	7.095	0.55400	0	0.00	0.000
02375	3	3	0.42	7.095	0.55400	0	0.00	0.000
02188	3	3	0.42	7.095	0.55400	0	0.00	0.000
02780	3	3	0.42	7.095	0.55400	0	0.01	0.000
02779	3	3	0.42	7.095	0.55400	0	0.00	0.000
02743	3	3	0.42	7.095	0.55400	0	0.00	0.000
02717	3	3	0.42	7.095	0.55400	0	0.00	0.000
02368	3	3	0.42	7.095	0.55400	0	0.01	0.000
02356	3	3	0.42	7.095	0.55400	0	0.00	0.000
02184	3	3	0.42	7.095	0.55400	0	0.01	0.000
02191	3	3	0.42	7.095	0.55400	0	0.00	0.000
02702	3	3	0.42	7.095	0.55400	0	0.00	0.000
02072	3	3	0.42	7.095	0.55400	0	0.01	0.000
02559	3	3	0.42	7.095	0.55400	0	0.00	0.000
02739	3	3	0.42	7.095	0.55400	0	0.00	0.000
02045	3	3	0.42	7.095	0.55400	0	0.00	0.000
02542	3	3	0.42	7.095	0.55400	0	0.00	0.000
02563	3	3	0.42	7.095	0.55400	0	0.00	0.000
02169	3	3	0.42	7.095	0.55400	0	0.01	0.000
02534	3	3	0.42	7.095	0.55400	0	0.00	0.000

02745	3	3	0.42	7.095	0.55400	0	0.01	0.000
02766	3	3	0.42	7.095	0.55400	0	0.01	0.000
02715	3	3	0.42	7.095	0.55400	0	0.00	0.000
02764	3	3	0.42	7.095	0.55400	0	0.00	0.000
02021	3	3	0.42	7.095	0.55400	0	0.01	0.000
02537	3	3	0.42	7.095	0.55400	0	0.00	0.000
02644	3	3	0.42	7.095	0.55400	0	0.00	0.000
02170	3	3	0.42	7.095	0.55400	0	0.01	0.000
02048	3	3	0.42	7.095	0.55400	0	0.01	0.000
02746	3	3	0.42	7.095	0.55400	0	0.00	0.000
02556	3	3	0.42	7.095	0.55400	0	0.00	0.000
02067	3	3	0.42	7.095	0.55400	0	0.00	0.000
02186	3	3	0.42	7.095	0.55400	0	0.01	0.000
02720	3	3	0.42	7.095	0.55400	0	0.01	0.000
02171	3	3	0.42	7.095	0.55400	0	0.00	0.000
02726	3	3	0.42	7.095	0.55400	0	0.00	0.000
02747	3	3	0.42	7.095	0.55400	0	0.01	0.000
02719	3	3	0.42	7.095	0.55400	0	0.00	0.000
02740	3	3	0.42	7.095	0.55400	0	0.01	0.000
02035	3	3	0.42	7.095	0.55400	0	0.00	0.000
02668	3	3	0.42	7.095	0.55400	0	0.00	0.000
02122	3	3	0.42	7.095	0.55400	0	0.01	0.000
02126	3	3	0.42	7.095	0.55400	0	0.01	0.000
02124	3	3	0.42	7.095	0.55400	0	0.01	0.000
02032	3	3	0.42	7.095	0.55400	0	0.00	0.000
02744	3	3	0.42	7.095	0.55400	0	0.00	0.000
02062	3	3	0.42	7.095	0.55400	0	0.01	0.000
02648	3	3	0.42	7.095	0.55400	0	0.00	0.000
02136	3	3	0.42	7.095	0.55400	0	0.01	0.000
02536	3	3	0.42	7.095	0.55400	0	0.01	0.000
02125	3	3	0.42	7.095	0.55400	0	0.01	0.000
02769	3	3	0.42	7.095	0.55400	1	0.00	344.335
02657	3	3	0.42	7.095	0.55400	0	0.00	0.000
02723	3	3	0.42	7.095	0.55400	0	0.00	0.000
02121	3	3	0.42	7.095	0.55400	2	0.01	277.864
