# Supplementary material for: A SaTScan™ macro accessory for cartography (SMAC) package implemented with SAS® software
Source: Int J Health Geogr. 2007 Mar 6;6:6. doi: 10.1186/1476-072X-6-6 (PMC1821006; doi:10.1186/1476-072X-6-6)
Supplement: Additional File 4 — SAS code for inputdata macro. This section contains the SAS code for the inputdata macro described in this paper. [file 1476-072X-6-6-S4.pdf]

```
%macro inputdata(datadir=, signaldate=, ndays=, coordinates_sas=,
coordinates_text=, cases_sas=, cases_text=, population_sas=,
population_text=);

libname a "&datadir";

%put; %put;
/* Check for the existence of &zipds SAS data set.  If it does not
exist, write an error message in the log and exit the macro */
%if %sysfunc(exist(a.&coordinates_sas))=0 %then %do;
      %put ERROR: The SAS data set "a.&coordinates_sas", which should
contain the location IDs, does not exist.  The INPUTDATA macro will
stop running.;
      %goto exit;
%end;

/* If the &zipds data set does exist, check to make sure that the 3
required variables exist.  If any of them are missing, write an error
message in the log and exit the macro */
%else %do;
      %let zipck=%sysfunc(open(a.&coordinates_sas,i));
      %if %sysfunc(varnum(&zipck,x))=0   %then %put ERROR: The required
variable "x", which should contain the longitude, was not found in the
data set "a.&coordinates_sas".  The INPUTDATA macro will stop running.;
      %if %sysfunc(varnum(&zipck,y))=0   %then %put ERROR: The required
variable "y", which should contain the latitude, was not found in the
data set "a.&coordinates_sas".  The INPUTDATA macro will stop running.;
      %if %sysfunc(varnum(&zipck,zip))=0 %then %put ERROR: The required
variable "zip", which should contain the location ID, was not found in
the data set "a.&coordinates_sas".  The INPUTDATA macro will stop
running.;
      %if (%sysfunc(varnum(&zipck,x))=0 or %sysfunc(varnum(&zipck,y))=0
or %sysfunc(varnum(&zipck,zip))=0) %then %do;
            %let close=%sysfunc(close(&zipck));
            %goto exit;
      %end;
%end;
%let close=%sysfunc(close(&zipck));


/* Check for the existence of &popds SAS data set.  If it does not
exist, write an error message in the log and exit the macro */
%if %sysfunc(exist(a.&population_sas))=0 %then %do;
      %put ERROR: The SAS data set "a.&population_sas", which should
contain the population for each location ID/date pair, does not exist.
The INPUTDATA macro will stop running.;
      %goto exit;
%end;

/* If the &popds data set does exist, check to make sure that the 3
required variables exist.  If any of them are missing, write an error
message in the log and exit the macro */
%else %do;
      %let popck=%sysfunc(open(a.&population_sas,i));
      %if %sysfunc(varnum(&popck,pop))=0  %then %put ERROR: The
required variable "pop", which should contain the population, was not
```

```
found in the data set "a.&population_sas".  The INPUTDATA macro will
stop running.;
      %if %sysfunc(varnum(&popck,zip))=0  %then %put ERROR: The
required variable "zip", which should contain the location ID, was not
found in the data set "a.&population_sas".  The INPUTDATA macro will
stop running.;
      %if (%sysfunc(varnum(&popck,pop))=0 or
%sysfunc(varnum(&popck,zip))=0) %then %do;
            %let close=%sysfunc(close(&popck));
            %goto exit;
      %end;
      %let popdate=%sysfunc(varnum(&popck,date));
      %if (&popdate=0) %then %put WARNING: The required variable "date"
was not found in the data set "a.&population_sas".  It will be assumed
that the population is the same every day in each location ID.;
%end;
%let close=%sysfunc(close(&popck));


/* Check for the existence of &caseds SAS data set.  If it does not
exist, write an error message in the log and exit the macro */
%if %sysfunc(exist(a.&cases_sas))=0 %then %do;
      %put ERROR: The SAS data set "a.&cases_sas", which should contain
the number of cases for each location ID/date pair, does not exist.
The INPUTDATA macro will stop running.;
      %goto exit;
%end;

/* If the &caseds data set does exist, check to make sure that the 3
required variables exist.  If any of them are missing, write an error
message in the log and exit the macro */
%else %do;
      %let caseck=%sysfunc(open(a.&cases_sas,i));
      %if %sysfunc(varnum(&caseck,count))=0 %then %put ERROR: The
required variable "count", which should contain the number of cases,
was not found in the data set "a.&cases_sas".  The INPUTDATA macro will
stop running.;
      %if %sysfunc(varnum(&caseck,date))=0  %then %put ERROR: The
required variable "date" was not found in the data set "a.&cases_sas".
The INPUTDATA macro will stop running.;
      %if %sysfunc(varnum(&caseck,zip))=0   %then %put ERROR: The
required variable "zip", which should contain the location ID, was not
found in the data set "a.&cases_sas".  The INPUTDATA macro will stop
running.;
      %if (%sysfunc(varnum(&caseck,count))=0 or
%sysfunc(varnum(&caseck,date))=0 or %sysfunc(varnum(&caseck,zip))=0)
%then %do;
            %let close=%sysfunc(close(&caseck));
            %goto exit;
      %end;
%end;
%let close=%sysfunc(close(&caseck));


/*  Now run the macro if there were no errors  */
data dates;
      startdate = input("&signaldate", mmddyy10.)-&ndays+1;
```

```
      enddate = input("&signaldate", mmddyy10.);
      format startdate enddate mmddyy10.;
run;
proc print data=dates noobs;  run;

%if &popdate=0 %then %do;
      proc sort data=a.&population_sas; by zip descending pop; run;
      data population;
            set a.&population_sas;
            by zip;
            if first.zip;
            do i=input("&signaldate", mmddyy10.)-&ndays to
input("&signaldate", mmddyy10.);
                  date=i;
                  output;
            end;
      run;
%end;
%else %if &popdate gt 0 %then %do;
      data population;
            set a.&population_sas;
      run;
%end;

proc sort data=a.&coordinates_sas; by zip; run;
proc sort data=population; by zip; run;
data zips;                    * Make the zip code file for SaTScan;
      merge population(in=keeppop where=(date=input("&signaldate",
mmddyy10.))) a.&coordinates_sas(in=location);
      by zip;
      if keeppop and location;
      file "&coordinates_text..txt";
      put zip y x;
run;

/* Check for the existence of the location file.  If it does not exist,
write an error message in the log and exit the macro */
%if %sysfunc(fileexist(&coordinates_text..txt))=0 %then %do;
      %put ERROR: The file "&coordinates_text..txt", which should
contain the location information for SaTScan, was not created.  Check
to see if the directory exists.  The INPUTDATA macro will stop
running.;
      %goto exit;
%end;

proc sort data=a.&cases_sas; by zip; run;
data _null_;                  * Make the case file for SaTScan;
      merge a.&cases_sas(in=case) zips(in=location keep=zip);
      by zip;
      if case and location and input("&signaldate", mmddyy10.)-&ndays
lt date le input("&signaldate", mmddyy10.);
      if (vtype(zip)='N' and zip ne .) or (vtype(zip)='C' and zip ne '
');
      if vtype(count)='N' and count=. then count=0;
      else if vtype(count)='C' and count=' ' then count='0';
      file "&cases_text..txt";
      put zip count date yymmddS10.;
```

```
run;

/* Check for the existence of the case file.  If it does not exist,
write an error message in the log and exit the macro */
%if %sysfunc(fileexist(&cases_text..txt))=0 %then %do;
      %put ERROR: The file "&cases_text..txt", which should contain the
case information for SaTScan, was not created.  Check to see if the
directory exists.  The INPUTDATA macro will stop running.;
      %goto exit;
%end;

data _null_;                  * Make the population file for SaTScan;
      merge population(in=keeppop) zips(in=location keep=zip);
      by zip;
      if keeppop and location and input("&signaldate", mmddyy10.)-
&ndays lt date le input("&signaldate", mmddyy10.);
      file "&population_text..txt";
      put zip date yymmddS10. ' ' pop;
run;

/* Check for the existence of the location file.  If it does not exist,
write an error message in the log and exit the macro */
%if %sysfunc(fileexist(&population_text..txt))=0 %then %do;
      %put ERROR: The file "&population_text..txt", which should
contain the population information for SaTScan, was not created.  Check
to see if the directory exists.  The INPUTDATA macro will stop
running.;
      %goto exit;
%end;
%exit: %mend inputdata;
```
