# Supplementary material for: A SaTScan™ macro accessory for cartography (SMAC) package implemented with SAS® software
Source: Int J Health Geogr. 2007 Mar 6;6:6. doi: 10.1186/1476-072X-6-6 (PMC1821006; doi:10.1186/1476-072X-6-6)
Supplement: Additional File 5 — SAS code for parameters macro. This section contains the SAS code for the parameters macro described in this paper. [file 1476-072X-6-6-S5.pdf]

```
%macro parameters(datastreams=, cases_text=, control_text=,
population_text=, coordinates_text=, usegridfile=, gridfile=,
timeprecision=, coordinatestype=, startdate=, enddate=, analysistype=,
modeltype=, scanareas=, timeaggunits=, timeagglength=, mcreps=,
results_text=, llr_ascii=, llr_dbase=, rr_ascii=, rr_dbase=,
gis_dbase=, cluster_dbase=, mdspurpose=, casefile2=, controlfile2=,
popfile2=, maxgeosize=, includepurelytemp=, maxspatialinterp=,
maxcirclepopfile=, maxtempsize=,  includepurelyspatial=,
maxtemporalinterp=, includeclusters=, intervalstartrange=,
intervalendrange=, timetrendadjtype=, timetrendpercent=,
adjustmentsbyrrfile=, useadjustmentbyrrfile=, spatialadjtype=,
prospectivestartdate=, earlytermination=, adjustearly=,
secondarycriteria=, reportmaxgeosize=, usereportmaxgeosize=,);

data prms;
   length value $135.;

   * Input;
   value='[Input]'; output;
   value="CaseFile=&cases_text..txt"; output;   * Case file;
   %if &modeltype=1 %then %do;
      value="ControlFile=&control_text..txt"; output; * Control file -
only needed when using Bernoulli model;
   %end;
   %else %if &modeltype=0 %then %do;
      value="PopulationFile=&population_text..txt"; output; *
Population file - only needed when using Poisson model;
   %end;
   value="CoordinatesFile=&coordinates_text..txt"; output;  * File
containing coordinates of zip code centroids;
   value="UseGridFile=&usegridfile"; output;
   value="GridFile=&gridfile"; output;
   value="PrecisionCaseTimes=&timeprecision"; output; * 0=None, 1=Year,
2=Month, 3=Day;
   value="CoordinatesType=&coordinatestype"; output;  * 0=Cartesian,
1=Lat/Long;
   value="StartDate=&startdate"; output;
   value="EndDate=&enddate"; output;
   value=" "; output;

   * Analysis;
   value='[Analysis]'; output;
   value="AnalysisType=&analysistype"; output;  * 1=Purely Spatial,
2=Purely Temporal, 3=Retrospective Space-Time, 4=Prospective Space-
Time, 5=N/A, 6=Prospective Purely Temporal;
   value="ModelType=&modeltype"; output;  * 0=Poisson, 1=Bernoulli,
2=Space-Time Permutation;
   value="ScanAreas=&scanareas"; output;  * Scan for areas with: 1=High
Rates, 2=Low Rates, 3=High or Low Rates;
   value="TimeAggregationUnits=&timeaggunits"; output;      * 0=None,
1=Year, 2=Month, 3=Day;
   value="TimeAggregationLength=&timeagglength"; output;    * positive
integer;
   value="MonteCarloReps=&mcreps"; output;
   value=" "; output;

   * Output;
```

```
   value='[Output]'; output;
   value="ResultsFile=&results_text..txt"; output;
   value="SaveSimLLRsASCII=&llr_ascii"; output;
   value="SaveSimLLRsDBase=&llr_dbase"; output;
   value="IncludeRelativeRisksCensusAreasASCII=&rr_ascii"; output;
   value="IncludeRelativeRisksCensusAreasDBase=&rr_dbase"; output;
   value="CensusAreasReportedClustersASCII=y"; output;
   value="CensusAreasReportedClustersDBase=&gis_dbase"; output;
   value="MostLikelyClusterEachCentroidASCII=y"; output;
   value="MostLikelyClusterEachCentroidDBase=&cluster_dbase"; output;
   value=" "; output;

%if "&datastreams" gt 1 %then %do; * Multiple Data Sets;
   value='[Multiple Data Sets]'; output;
   value="MultipleDataSetsPurposeType=&mdspurpose"; output; *
0=multivariate, 1=adjustment;
   value="CaseFile2=&casefile2..txt"; output;   * case data set 2;
   %if &modeltype=1 %then %do;
      value="ControlFile2=&controlfile2..txt"; output;      * control
data 2;
   %end;
   %else %if &modeltype=1 %then %do;
      value="PopulationFile2=&popfile2..txt"; output; * population data
2;
   %end;
   value=" "; output;
%end;

   * Spatial Window;
   value='[Spatial Window]'; output;
   value="MaxGeographicSize=&maxgeosize"; output;     * <=50%;
   value="IncludePurelyTemporal=&includepurelytemp"; output;
   value="MaxSpatialSizeInterpretation=&maxspatialinterp"; output;
   * 0=Percentage, 1=Distance, 2=Percentage of max circle population
file;
   value="MaxCirclePopulationFile=&maxcirclepopfile"; output;     *
maximum circle size filename;
   value=" "; output;

   * Temporal Window;
   value='[Temporal Window]'; output;
   value="MaxTemporalSize=&maxtempsize"; output;      * <=90%;
   value="IncludePurelySpatial=&includepurelyspatial"; output;
   value="MaxTemporalSizeInterpretation=&maxtemporalinterp"; output;
   * 0=Percentage, 1=Time;
   value="IncludeClusters=&includeclusters"; output;  * clusters to
include: 0=All, 1=Alive, 2=Flexible Window;
   value=" "; output;

   * Space and Time Adjustments;
   value='[Space and Time Adjustments]'; output;
   value="IntervalStartRange=&intervalstartrange"; output;  * flexible
temporal window start range (YYYY/MM/DD,YYYY/MM/DD);
   value="IntervalEndRange=&intervalendrange"; output;      * flexible
temporal window end range (YYYY/MM/DD,YYYY/MM/DD);
```

```
   value="TimeTrendAdjustmentType=&timetrendadjtype"; output;
   * 0=None, 1=Nonparametric, 2=LogLinearPercentage,
3=CalculatedLogLinearPercentage, 4=TimeStratifiedRandomization;
   value="TimeTrendPercentage=&timetrendpercent"; output;   * time
trend adjustment percentage (>-100);
   value="AdjustmentsByKnownRelativeRisksFilename=&adjustmentsbyrrfile"
; output;   * adjustments by known relative risks file name (with HA
Randomization=1 or ...);
   value="UseAdjustmentsByRRFile=&useadjustmentbyrrfile"; output; * use
adjustments by known relative risks file? (y/n);
   value="SpatialAdjustmentType=&spatialadjtype"; output;   * 0=no
spatial adjustment, 1=spatially stratified randomization;
   value=" "; output;

   * Inference;
   value='[Inference]'; output;
   if "&adjustearly"='n' then do;
      value="ProspectiveStartDate=&enddate"; output;
   end;     * prospective surveillance start date (YYYY/MM/DD);
   else if "&adjustearly"='y' then do;
      value="ProspectiveStartDate=&prospectivestartdate"; output;
   end;     * prospective surveillance start date (YYYY/MM/DD);
   value="EarlySimulationTermination=&earlytermination"; output;
   value="AdjustForEarlierAnalyses=&adjustearly"; output;   *
Prospective only (y/n);
   value=" "; output;

   * Clusters Reported;
   value='[Clusters Reported]'; output;
   value="CriteriaForReportingSecondaryClusters=&secondarycriteria";
output;     * 0=NoGeoOverlap, 1=NoCentersInOther,
2=NoCentersInMostLikely, 3=NoCentersInLessLikely,
4=NoPairsCentersEachOther, 5=NoRestrictions;
   value="MaxReportedGeoClusterSize=&reportmaxgeosize"; output; * < max
geographical cluster size %;
   value="UseReportOnlySmallerClusters=&usereportmaxgeosize"; output;
   value=" "; output;

   * Batch Mode Features;
   value='[BatchMode Features]'; output;
   value='ValidateParameters=y'; output;
   value=" "; output;

   * System Features;
   value='[System]'; output;
   value='; system setting - do not modify'; output;
   value='Version=5.1.0'; output;
   value=' '; output;

run;

data _null_;
   set prms;
   file "&satscandir.\parameterfile.prm"; * Save the parameter file in
the SaTScan directory;
   put value;
run;
```

```
/* Check for the existence of the parameter file.  If it does not
exist, write an error message in the log */
%if %sysfunc(fileexist(&satscandir.\parameterfile.prm))=0 %then %do;
   %put ERROR: The file "&satscandir.\parameterfile.prm", which should
contain the parameter file for SaTScan, does not exist.;
%end;

data _null_;
   set prms;
   file "&results_text..prm"; * Save the parameter file in the SaTScan
directory;
   put value;
run;
/* Check for the existence of the parameter file.  If it does not
exist, write an error message in the log */
%if %sysfunc(fileexist(&results_text..prm))=0 %then %do;
   %put WARNING: The file "&results_text..prm", which should contain
the parameter file for SaTScan, does not exist.;
%end;

%exit: %mend parameters;
```
