# Supplementary material for: A SaTScan™ macro accessory for cartography (SMAC) package implemented with SAS® software
Source: Int J Health Geogr. 2007 Mar 6;6:6. doi: 10.1186/1476-072X-6-6 (PMC1821006; doi:10.1186/1476-072X-6-6)
Supplement: Additional File 6 — SAS code for map macro. This section contains the SAS code for the map macro described in this paper. [file 1476-072X-6-6-S6.pdf]

```
%macro map(library=, uszips=, zipbounds=, results_text=, mapfile=,
   studystart=, studyend=);

libname maps "&library";
%annomac;

/* Check for the existence of the txt file.  If it does not exist,
   write an error message in the log */
%if %sysfunc(fileexist(&results_text..txt))=0 %then %do;
   %put ERROR: The file "&results_text..txt", which should contain the
   information about the SaTScan cluster, does not exit.  The MAP macro
   will stop running.;
   %goto exit;
%end;

/* Check for the existence of the col file.  If it does not exist,
   write an error message in the log */
%if %sysfunc(fileexist(&results_text..col.txt))=0 %then %do;
   %put ERROR: The file "&results_text..col.txt", which should contain
   the information about the SaTScan cluster, does not exit.  The MAP
   macro will stop running.;
   %goto exit;
%end;

/* Check for the existence of the gis file.  If it does not exist,
   write an error message in the log */
%if %sysfunc(fileexist(&results_text..gis.txt))=0 %then %do;
   %put ERROR: The file "&results_text..gis.txt", which should contain
   the location information about the SaTScan cluster, does not exit.
   The MAP macro will stop running.;
   %goto exit;
%end;

data center1;     * Read the signal information from SaTScan;
   infile "&results_text..col.txt";
   input @1 zip$5. @33 cluster @39 y @52 x @65 radius @78 nzips @91
   observed @104 expected @117 RR @130 LLR @142 pvalue @155 startdate
   yymmdd10. @173 enddate yymmdd10.;
   if cluster=1;
   format startdate enddate mmddyy10.;
run;

proc sort data=maps.&uszips; by zip; run;
data center;
   merge center1(in=keep) maps.&uszips;
   by zip;
   if keep;
run;

data tempcluster; * Read in the zip codes that are in the signal;
   infile "&results_text..gis.txt";
   input @1 zip$5. @33 cluster;
   if cluster=1;
   if _n_=1 then color=1;
   else color=2;
   drop cluster;
run;
```

```
data temp; set maps.&zipbounds; run; * Merge with zip code centers so
   that all zips will show up on the map;
proc sort data=temp out=ziplist nodupkey; by zip;     run;
proc sort data=tempcluster; by zip; run;
data cluster;
   merge tempcluster ziplist;
   by zip;
   if color = . then color=0;
   drop x y region i;
run;

data anno1;       * Make an annotate data set to put center, radius,
   observed and expected cases, and p-value on the map;
   set center;
   length text $80.;
   retain hsys '4' xsys '3' ysys '3' when 'a' style 'swiss' color 'red'
   position '6' function 'label';
   ndays = enddate - startdate + 1;
   start = put(mdy(scan("&studystart",2,'/'),
   scan("&studystart",3,'/'), scan("&studystart",1,'/')), mmddyy10.);
   end = put(mdy(scan("&studyend",2,'/'), scan("&studyend",3,'/'),
   scan("&studyend",1,'/')), mmddyy10.);
   * Makes labels;
   x=2; y=99.5;   text='Study Period: '||start||' to '||end; output;
   x=2; y=96.5;   text='Cluster dates: '||put(startdate, mmddyy10.)||'
   to '||put(enddate, mmddyy10.)||' ('||compress(ndays)||' days)';
   output;
   x=2; y=93.5;   text='Centered in '||compress(city)||',
   '||compress(state); output;
   x=2; y=90.5;   text='Center='||compress(zip); output;
   x=22; y=90.5;  text='Radius='||compress(radius)||' km'; output;
   x=2; y=87.5;   text='Observed cases='||compress(observed); output;
   x=22; y=87.5;  text='Expected cases='||compress(expected); output;
   x=42; y=87.5;  text='P-value='||compress(pvalue); output;
run;

data anno2; * Make an annotate data set to put list of zip codes on the
   map;
   set cluster(rename=(color=c));
   length text $80.;
   retain hsys '4' xsys '3' ysys '3' when 'a' style 'swiss' color
   'black' position '6' function 'label';
   where c ne 0;
   if _n_=1 then do;
      x=2;  y=45; text='Zip codes in cluster:'; output; end;
   if _n_ le 11 then do;
      x=2+(_n_-1)*5;    y=42; text=zip;   output; end;
   else if _n_ le 22 then do;
       x=2+(_n_-12)*5;  y=39; text=zip;   output; end;
   else if _n_ le 33 then do;
       x=2+(_n_-23)*5;  y=36; text=zip;   output; end;
   else if _n_ le 44 then do;
       x=2+(_n_-34)*5;  y=33; text=zip;   output; end;
   else if _n_ le 55 then do;
      x=2+(_n_-45)*5;   y=30; text=zip;   output; end;
   else if _n_ le 66 then do;
```

```
      x=2+(_n_-56)*5;   y=27; text=zip;   output; end;
   else if _n_ le 77 then do;
      x=2+(_n_-67)*5;   y=24; text=zip;   output; end;
   else if _n_ le 88 then do;
      x=2+(_n_-78)*5;   y=21; text=zip;   output; end;
   else if _n_ le 99 then do;
      x=2+(_n_-89)*5;   y=18; text=zip;   output; end;
   else if _n_ le 110 then do;
      x=2+(_n_-100)*5;  y=15; text=zip;   output; end;
   else if _n_ le 121 then do;
      x=2+(_n_-111)*5;  y=12; text=zip;   output; end;
   else if _n_ le 132 then do;
      x=2+(_n_-122)*5;  y=9;  text=zip;   output; end;
   else if _n_ le 143 then do;
      x=2+(_n_-133)*5;  y=6;  text=zip;   output; end;
   else if _n_ le 154 then do;
      x=2+(_n_-144)*5;  y=3;  text=zip;   output; end;
   else if _n_ gt 154 then do;
      x=62; y=3;  text='See output for other zip codes'; output; end;
run;

data anno; length color $5. text $80.; set anno1 anno2; run;      *
   This is the annotate data set to be used on the map.  It writes the
   center of the signal, the radius, the city where the signal is
   centered, the observed and expected cases, the p-value, and a list
   of the zip codes included in the signal;

goptions reset=all targetdevice=cgmof97l device=cgmof97l gsfname=map
   gsfmode=replace;

pattern1 color=white repeat=1;
pattern2 color=red repeat=1;
pattern3 color=gray repeat=1;

filename map "&mapfile..cgm";
proc gmap map=maps.&zipbounds data=cluster;     * Draw the map.
   maps.&zipbounds is the data set containing the boundary information
   about the zip codes;
   id zip;
   choro color/nolegend discrete coutline=ltgray annotate=anno;
run; quit;
goptions reset=all;

/* Check for the existence of the cgm map file.  If it does not exist,
   write an error message in the log */
%if %sysfunc(fileexist(&mapfile..cgm))=0 %then %do;
   %put ERROR: The file "&mapfile..cgm", which should contain a map of
   the most likely SaTScan cluster, was not created.;
   %goto exit;
%end;

%exit: %mend map;
```
