# Supplementary material for: A SaTScan™ macro accessory for cartography (SMAC) package implemented with SAS® software
Source: Int J Health Geogr. 2007 Mar 6;6:6. doi: 10.1186/1476-072X-6-6 (PMC1821006; doi:10.1186/1476-072X-6-6)
Supplement: Additional File 7 — SAS code for SMAC macro. This section contains the SAS code for the SMAC macro described in this paper. [file 1476-072X-6-6-S7.pdf]

```
%macro SMAC(macrodir=, datadir=, satscandir=C:\Program Files\SaTScan,
date=, ndays=100, datastreams=1, coordinates_sas=, coordinates_text=,
cases_sas=, cases_text=, population_sas=MA_zipcode_population,
population_text=, control_text=, gridfile=, usegridfile=n,
timeprecision=3, coordinatestype=1, analysistype=4, modeltype=0,
scanareas=1, timeaggunits=3, timeagglength=1, mcreps=999,
results_text=, llr_ascii=n, llr_dbase=n, rr_ascii=n, rr_dbase=n,
gis_dbase=n, cluster_dbase=n, mdspurpose=, cases2=, controlfile2=,
pop2=, maxgeosize=50, includepurelytemp=n, maxspatialinterp=0,
maxcirclepopfile=, maxtempsize=3, includepurelyspatial=n,
maxtemporalinterp=1, includeclusters=0,
intervalstartrange=%str(2000/1/1,2000/12/31),
intervalendrange=%str(2000/1/1,2000/12/31), timetrendadjtype=0,
timetrendpercent=0.000000, adjustmentsbyrrfile=,
useadjustmentbyrrfile=n, spatialadjtype=0, prospectivestartdate=,
earlytermination=y, adjustearly=y, secondarycriteria=0,
reportmaxgeosize=50, usereportmaxgeosize=n, uszips=uszips,
zipbounds=mazips, map=);

%let mapslibrary=&macrodir.\maps;

%include "&macrodir\Macro 1 - Make files for SaTScan.sas";
%include "&macrodir\Macro 3 - Map SaTScan output.sas";
%include "&macrodir\Macro 4 - Make parameter file for SaTScan.sas";


%inputdata(datadir=&datadir, signaldate=&date, ndays=&ndays,
coordinates_sas=&coordinates_sas, coordinates_text=&coordinates_text,
cases_sas=&cases_sas, cases_text=&cases_text,
population_sas=&population_sas, population_text=&population_text);

/* Check for the existence of the case file.  If it does not exist,
write an error message in the log and exit the macro */
%if %sysfunc(fileexist(&cases_text..txt))=0 %then %do;
      %put ERROR: The file "&cases_text..txt", which should contain the
case information for SaTScan, does not exist.  The PARAMETERS macro,
SaTScan, and the MAP macro will not run.;
      %goto exit;
%end;

/* Check for the existence of the population file.  If it does not
exist, write an error message in the log and exit the macro */
%if (&modeltype=0 and %sysfunc(fileexist(&population_text..txt))=0)
%then %do;
      %put ERROR: The file "&population_text..txt", which should
contain the population information for SaTScan, does not exist.  The
PARAMETERS macro, SaTScan, and the MAP macro will not run.;
      %goto exit;
%end;

/* Check for the existence of the control file.  If it does not exist,
write an error message in the log and exit the macro */
%if (&modeltype=1 and %sysfunc(fileexist(&control_text..txt))=0) %then
%do;
      %put ERROR: The file "&control_text..txt", which should contain
the control information for SaTScan, does not exist.  The PARAMETERS
macro, SaTScan, and the MAP macro will not run.;
```

```
      %goto exit;
%end;

/* Check for the existence of the coordinates file.  If it does not
exist, write an error message in the log and exit the macro */
%if %sysfunc(fileexist(&coordinates_text..txt))=0 %then %do;
      %put ERROR: The file "&coordinates_text..txt", which should
contain the location information for SaTScan, does not exist.  The
PARAMETERS macro, SaTScan, and the MAP macro will not run.;
      %goto exit;
%end;


/* Check to make sure date range makes sense */
data _null_;
      set dates;
      call symput('start', put(startdate, yymmddS10.));
      call symput('end', put(enddate, yymmddS10.));
      call symput('datediff', enddate-startdate);
run;
%if &datediff < 0 %then %do;
      %put ERROR: The START date occurs after the END date.  The
PARAMETERS macro, SaTScan, and the MAP macro will not run.;
      %goto exit;
%end;


%parameters(datastreams=&datastreams,

            cases_text = &cases_text,
            control_text = &control_text,
            population_text = &population_text,
            coordinates_text = &coordinates_text,
            usegridfile = &usegridfile,
            gridfile = &gridfile,
            timeprecision = &timeprecision,
            coordinatestype = &coordinatestype,
            startdate = &start,
            enddate = &end,

            analysistype = &analysistype,
            modeltype = &modeltype,
            scanareas = &scanareas,
            timeaggunits = &timeaggunits,
            timeagglength = &timeagglength,
            mcreps = &mcreps,

            results_text = &results_text,
            llr_ascii = &llr_ascii,
            llr_dbase = &llr_dbase,
            rr_ascii = &rr_ascii,
            rr_dbase = &rr_dbase,
            gis_dbase = &gis_dbase,
            cluster_dbase = &cluster_dbase,

            mdspurpose=&mdspurpose,
            casefile2=&cases2,
```

```
            controlfile2=&controlfile2,
            popfile2=&pop2,

            maxgeosize = &maxgeosize,
            includepurelytemp = &includepurelytemp,
            maxspatialinterp = &maxspatialinterp,
            maxcirclepopfile = &maxcirclepopfile,

            maxtempsize = &maxtempsize,
            includepurelyspatial = &includepurelyspatial,
            maxtemporalinterp = &maxtemporalinterp,
            includeclusters = &includeclusters,

            intervalstartrange = &intervalstartrange,
            intervalendrange = &intervalendrange,
            timetrendadjtype = &timetrendadjtype,
            timetrendpercent = &timetrendpercent,
            adjustmentsbyrrfile = &adjustmentsbyrrfile,
            useadjustmentbyrrfile = &useadjustmentbyrrfile,
            spatialadjtype = &spatialadjtype,

            prospectivestartdate = &prospectivestartdate,
            earlytermination = &earlytermination,
            adjustearly = &adjustearly,

            secondarycriteria = &secondarycriteria,
            reportmaxgeosize = &reportmaxgeosize,
            usereportmaxgeosize = &usereportmaxgeosize);

/* Check for the existence of the parameter file.  If it does not
exist, write an error message in the log */
%if %sysfunc(fileexist(&satscandir.\parameterfile.prm))=0 %then %do;
      %put ERROR: The file "&satscandir.\parameterfile.prm", which
should contain the parameter file for SaTScan, does not exist.  SaTScan
will not run.;
      %goto exit;
%end;

x "cd &satscandir";     * Opens a command prompt window and changes to
the directory where SaTScan is stored;
x "SaTScanBatch.exe parameterfile.prm";   * Run SaTScan in batch mode;

/* Check for the existence of the txt file.  If it does not exist,
write an error message in the log */
%if %sysfunc(fileexist(&results_text..txt))=0 %then %do;
      %put ERROR: The file "&results_text..txt", which should contain
the information about the SaTScan cluster, was not created.  SaTScan
did not run successfully.  Run SaTScan manually with the parameter file
"&satscandir.\parameterfile.prm" to get SaTScan error messages.;
      %goto exit;
%end;

/* Check for the existence of the col file.  If it does not exist,
write an error message in the log */
%if %sysfunc(fileexist(&results_text..col.txt))=0 %then %do;
      %put ERROR: The file "&results_text..col.txt", which should
contain the information about the SaTScan cluster, was not created.
```

```
SaTScan did not run successfully.  Run SaTScan manually with the
parameter file "&satscandir.\parameterfile.prm" to get SaTScan error
messages.;
      %goto exit;
%end;

/* Check for the existence of the gis file.  If it does not exist,
write an error message in the log */
%if %sysfunc(fileexist(&results_text..gis.txt))=0 %then %do;
      %put ERROR: The file "&results_text..gis.txt", which should
contain the location information about the SaTScan cluster, was not
created.  SaTScan did not run successfully.  Run SaTScan manually with
the parameter file "&satscandir.\parameterfile.prm" to get SaTScan
error messages.;
      %goto exit;
%end;

%map(library=&mapslibrary, uszips=&uszips, zipbounds=&zipbounds,
results_text=&results_text, mapfile=&map, studystart=&start,
studyend=&end);

%exit: %mend SMAC;
```
