# Supplementary material for: A SaTScan™ macro accessory for cartography (SMAC) package implemented with SAS® software
Source: Int J Health Geogr. 2007 Mar 6;6:6. doi: 10.1186/1476-072X-6-6 (PMC1821006; doi:10.1186/1476-072X-6-6)
Supplement: Additional File 8 — Parameter file generated by parameters macro. This is an example of a typical parameter file generated from the parameters macro. [file 1476-072X-6-6-S8.pdf]

```
[Input]
CaseFile=c:\SMAC\SaTScan files\cases.txt
PopulationFile=c:\SMAC\SaTScan files\population.txt
CoordinatesFile=c:\SMAC\satscan files\zipcodes.txt
UseGridFile=n
GridFile=
PrecisionCaseTimes=3
CoordinatesType=1
StartDate=2002/03/12
EndDate=2002/09/09

[Analysis]
AnalysisType=4
ModelType=0
ScanAreas=1
TimeAggregationUnits=3
TimeAggregationLength=1
MonteCarloReps=999

[Output]
ResultsFile=c:\SMAC\SaTScan files\crypto output.txt
SaveSimLLRsASCII=n
SaveSimLLRsDBase=n
IncludeRelativeRisksCensusAreasASCII=n
IncludeRelativeRisksCensusAreasDBase=n
CensusAreasReportedClustersASCII=y
CensusAreasReportedClustersDBase=n
MostLikelyClusterEachCentroidASCII=y
MostLikelyClusterEachCentroidDBase=n

[Spatial Window]
MaxGeographicSize=50
IncludePurelyTemporal=n
MaxSpatialSizeInterpretation=0
MaxCirclePopulationFile=

[Temporal Window]
MaxTemporalSize=14
IncludePurelySpatial=n
MaxTemporalSizeInterpretation=1
IncludeClusters=0

[Space and Time Adjustments]
IntervalStartRange=2000/1/1,2000/12/31
IntervalEndRange=2000/1/1,2000/12/31
TimeTrendAdjustmentType=0
TimeTrendPercentage=0.000000
AdjustmentsByKnownRelativeRisksFilename=
UseAdjustmentsByRRFile=n
SpatialAdjustmentType=0

[Inference]
ProspectiveStartDate=2002/09/09
EarlySimulationTermination=y
AdjustForEarlierAnalyses=n

[Clusters Reported]
```

```
CriteriaForReportingSecondaryClusters=0
MaxReportedGeoClusterSize=50
UseReportOnlySmallerClusters=n

[BatchMode Features]
ValidateParameters=y

[System]
; system setting - do not modify
Version=5.1.0
```
