# Supplementary material for: A SaTScan™ macro accessory for cartography (SMAC) package implemented with SAS® software
Source: Int J Health Geogr. 2007 Mar 6;6:6. doi: 10.1186/1476-072X-6-6 (PMC1821006; doi:10.1186/1476-072X-6-6)
Supplement: Additional File 9 — SAS macro to run SMAC package. This is a sample SAS macro which can be used to run the SMAC package many times. [file 1476-072X-6-6-S9.pdf]

```
%macro manysatscans(startdate, lastdate);

data _null_;
   call symput('sdate', input("&startdate", mmddyy10.));
   call symput('ldate', input("&lastdate", mmddyy10.));
run;

%do i = &sdate %to &ldate;

   data _null_;
      day=day(&i);
      month=month(&i);
      year=year(&i);
      call symput('signaldate', compress(month||'/'||day||'/'||year));
      call symput('filedate', compress(month||'_'||day||'_'||year));
   run;

%SMAC(macrodir = c:\SMAC,
      datadir = c:\SMAC,
      satscandir=C:\Program Files\SaTScan\SaTScan 5.0,
      date = &signaldate,
      ndays=182,
      coordinates_sas = MAzipcentroids,
      coordinates_text = c:\SMAC\satscan files\&filedate. zipcodes,
      cases_sas = crypto_case_input_to_smac,
      cases_text = c:\SMAC\SaTScan files\&filedate. cases,
      population_sas = MA_zipcode_population,
      population_text = c:\SMAC\SaTScan files\&filedate. population,
      results_text = c:\SMAC\SaTScan files\&filedate. crypto output,
      map = c:\SMAC\maps\&filedate. crypto map,
      adjustearly = n,
      maxtempsize = 14);
%end;

%mend manysatscans;
```
